# Transactions of the Pathological Society of Philadelphia

**Published:** 1839-10-19

**Authors:** 


### TRANSACTIONS OF THE PATHOLOGICAL SOCIETY OF PHILADELPHIA.

*October* 14*th*, 1839.

The President, Dr. Gerhard, in the chair.

Dr. Stillé exhibited a specimen of *Fungoid Tumours of the Tibia*, and read the following notes of the case:

I. R., an Irishman by birth, a journeyman in a powder factory, and aged thirty-three years, was in the enjoyment of robust health, when, about twenty months ago, he fell, and struck the outer part of his left knee, between the tubercle of the tibia and the head of the fibula, upon a rounded stone. No ecchymosis followed the injury, but a small lump made its appearance within a few hours afterwards, which, after three or four months, increased to about the size of a hen's egg, and rendered the movements of the knee so difficult and painful as to oblige him to abandon his employment. He came to the Pennsylvania Hospital, where, after a mature examination, the tumour was thought to be aneurismal; and in September, 1838, the femoral artery was tied in the hope of curing it. During five weeks the operation seemed to have been successful, but at the end of that time the patient was discharged at his own request. On his return home he ceased to observe the precautions he had been advised to; and the tumour reappeared, and continued to increase slowly until May, 1839, when, on making a sudden effort to straighten his leg, he felt, to use his own expression, "something give way about the knee." From this period the growth of the tumour was more rapid; R. was unable to leave his bed, on account of its weight, and the pain which change of position produced in it, and he again entered the hospital during the last month. It increased notably during several weeks, and the surgeons of the institution decided that it should be removed by amputation of the thigh.

The following note was taken on the day preceding the operation:—The leg was bent upon the thigh at an angle of from 90° to 100°, and could be moved passively within these limits, but not without inflicting considerable pain. The muscles of the whole limb were emaciated; there was some œdema of the foot. The health of the patient was good, with the exception of a general feebleness referrible to his long confinement in bed. One of the glands of the left groin was slightly enlarged and sore. The tumour formed a rounded mass, commencing at eight inches and a half from the lower end of the tibia, and extending to the patella and the condyles of the femur, which could be felt as distinct from it. The skin covering it was a little more ruddy and smooth than that of the thigh, and was traversed by dilated and tortuous veins. The surface, in general, was smooth, and, although undulating, presented no abrupt prominences nor depressions. The tumour was of a more regularly rounded form in its anterior and external portions than internally and behind. Its greatest circumference measured sixteen inches. An inch above its junction with the tibia below it measured twelve inches; and on a line passing through the point of the patella and the ham, just above the tumour, twelve inches and a half in circumference. Its greatest length, or that measured from the point of the patella to the nearest point of the shaft of the tibia, was five inches. To the touch, the skin covering the tumour was sensibly warmer than on the thigh. It offered various degrees of hardness—its outer half being almost as resistant as bone, and somewhat elastic under firm pressure, which was painful. A like degree of hardness existed in the whole circumference of the tumour near its upper and lower lines of attachment,—but in the middle space, on its inner side, there was much less hardness, and a high degree of elasticity, but no signs of fluctuation. In the ham the tumour was soft, but less elastic. When the tumour was lightly grasped by the hands, a faint pulsation could be distinguished. The pulses of the poplitial artery could be felt high up in the ham, and at the same point there was a distinct *bruit de souffle*. Where the shaft of the tibia joined the tumour, an angle was formed, owing partly to the convexity of this latter, and partly to the fact that the axis of the tibia was not directed towards the condyles of the femur, but to a point behind them. All attempts to move the leg passively gave great pain.

The amputation of the thigh was performed by Dr. Norris on the 16th inst.; the femur was divided about the junction of its middle with its lower third. The medulla of the upper fragment was of a light yellow colour, and of a pasty consistence, and the bone was less resistant than natural. *Nineteen arteries* were secured at the time of the operation, and secondary hæmorrhage having occurred about ten hours afterwards, five additional ligatures had to be applied.

The excised portion of the femur being sawn in two through its epiphysis, the reticulated structure was found filled with a yellow, butter-like substance, and was easily crushed by the finger. The medullary canals of the femur and tibia were filled by the same yellow substance, and these bones themselves had a sensibly diminished consistence. On dissecting away from the tumour the skin and muscles, (which were pale and flabby,) its greatest circumference measured thirteen inches and a half. The cartilages of the knee-joint presented no unusual characters, except a thinning and slight discoloration of the one lining the external articulating fossa of the head of the tibia. The poplitial vessels and nerves lay over the back part of the tumour, and separated from it by their common sheath, but no branch from them could be found communicating with its interior. There was no appreciable deviation from the ordinary size and structure of the poplitial artery. Several tunics enveloped the tumour, viz. 1*st.* A complete one of condensed cellular substance, mingled with a little fat. 2*d.* The superficial fascia of the muscles of the upper part of the leg, considerably thickened, and apparently continuous with the periosteum. 3*d.* A bony shell entirely surrounding the tumour at its lower, and also at its upper part, but in the intermediate space complete on the outer half of the tumour alone, the inner half presenting only a few bony spicula distributed over its fibrous layer. This bony shell was from four to five lines thick where it joined the shaft of the tibia below, and the articular cartilages above; in other parts it was pretty uniformly about two or three lines in thickness. It appeared to consist in a globular expansion of the external table of the tibia, while the internal table, the intermediate spongy structure, and the medulla, were continuous with the cavity of the tumour, and the matter contained in it. This matter was chiefly composed of a soft solid, closely resembling a child's brain that had been crushed by the fingers. It was of a ruddy or brownish colour, and generally disposed around a number of central masses or nuclei, which had in some places the characters of yellow, semi-transparent coagula,—and in others of a cerebriform matter, rather firmer than the more superficial portions of the tumour. In the centre of one nucleus of the latter sort, a few grains of calcareous matter were detected. Under the spot formerly occupied by the supposed aneurismal tumour, but within the present bony parietes, was found a clot about two inches and a half long, by three-quarters of an inch thick, of a buff colour, lamellated in its structure, and firm under pressure. It was surrounded on every side by the prevailing brain, like matter of the cavity. A number of rounded apertures might be perceived on the surface of a section of the former portions of the tumour, but their walls were very easily torn by the probe. No vessels could be distinctly traced within the tube, but streaks, and small masses of blood, were frequently to be met with. Several small cysts were opened, and found to contain a yellowish, transparent, viscid fluid.

*Recapitulation.*

A man aged 33 years, in robust health, receives a blow upon the head of the tibia; a small tumour appears there, which increases slowly for six months, when, presenting the usual characters of an aneurism, the femoral artery is tied, with a view of curing it, but without permanent success. The tumour augments in volume, till it surrounds the upper fourth of the leg; the thigh is amputated, and the tumour is then found to consist in an expansion of the upper fourth of the shaft of the tibia,—to be apparently unconnected in a direct manner with the poplitial vessels,—to contain, for the most part, a substance like softened brain, besides several clots, one of which, and the oldest, occupies the seat of the original pulsating tumour.

*Remarks.*

It would seem probable that the disease, in the case before us, commenced in the medulla of the tibia, since, in the parts adjacent to the tumour in process of change, the medulla is clearly the tissue most altered in all its characters. The difficulty of accounting for the pulsations and other signs of aneurism in the original tumour, should be remembered, in order that future observers may determine in what manner tumours like the present are supplied with blood. It is worthy of notice that there was no vestige of scirrhus in this tumour, (recently formed as it was,) a fact at war with the opinion that the cerebriform matter is always the result of softening in a scirrhous mass. Is it not probable, considering the state of the medulla in the upper portion of the divided femur, that the disease will be reproduced? and, if so, under what form will it reappear? I am not aware that pathologists have afforded any data which may enable us to answer the latter question.

### THE MEDICAL EXAMINER.

PHILADELPHIA, OCTOBER 19, 1839.

